# Low-Carbohydrate High-Fat Diets, Lipid Levels, and Cardiovascular Risk∗

**DOI:** 10.1016/j.jacadv.2024.100975

**Published:** 2024-05-15

**Authors:** Moriah P. Bellissimo, W. Gregory Hundley

**Affiliations:** Division of Cardiology, Pauley Heart Center, Virginia Commonwealth University School of Medicine, Richmond, Virginia, USA

**Keywords:** atherogenic, cardiac, cholesterol, ketogenic diet, nutrition

**Figure:**
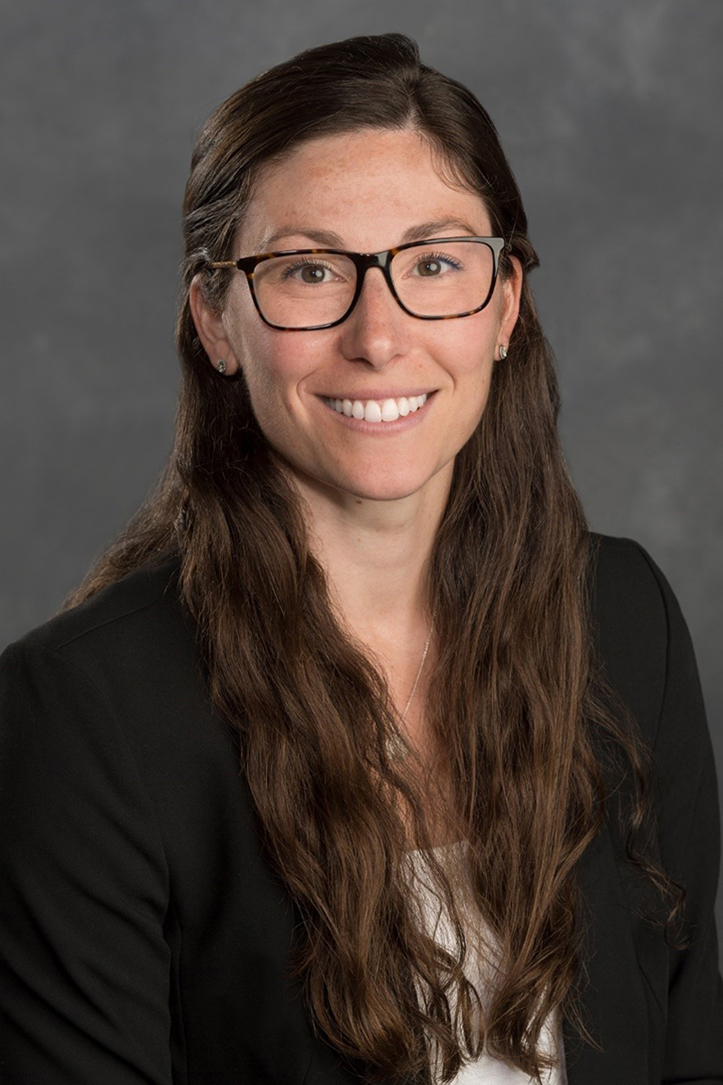

Dietary intake is a leading risk factor for cardiovascular diseases (CVD),^1^ thus, an important factor to consider when selecting a dietary pattern is its impact on cardiovascular (CV) health and function. One dietary pattern that cycles in popularity is a low-carbohydrate, high-fat (LCHF) diet. In the 1970s, this was popularized as the Atkins diet,^2^ and one of its most recent iterations is the Paleo diet. There is concern these LCHF diets may promote an atherogenic lipid profile that increases CVD risk, but data are limited in this area.

This issue of *JACC: Advances* features a study by Iatan et al^3^ which investigated the association between an LCHF dietary pattern with serum lipid levels and incident major adverse cardiovascular events (MACE). Genetic variants associated with hypercholesterolemia were characterized to test interindividual response to an LCHF diet. To accomplish this work, the authors used data from the UK Biobank cohort. Participants completed a validated self-administered, web-based 24-hour dietary recall. An LCHF diet was defined as reporting <100 g of carbohydrates per day and/or <25% total daily energy from carbohydrates and >45% total daily energy from fat. Individuals not meeting these criteria were classified as consuming a standard diet (SD). The whole cohort completed a 24-hour dietary recall at various time points in the study. LCHF diet participants (n = 2,034) were age- and sex-matched (4:1) to 8,136 control participants reporting an SD. Incident MACE or composite atherosclerotic cardiovascular disease (ASCVD) were noted by evaluating hospital admission and International Classification of Diseases-10th revision, coding in electronic health records. To investigate a temporal association, a subset of the cohort was identified who completed a 24-hour dietary recall and blood testing at the initial study visit (LCHF, n = 305; SD, n = 1,220) and followed overtime. Participants were also categorized as having or not having a high low-density-lipoprotein cholesterol (LDL-C) polygenic risk score (PRS) for familial hypercholesterolemia-causing variants.

Findings were similar in the whole cohort and subset of the cohort with diet and lipids assessed concomitantly. The LCHF diet group exhibited higher levels of LDL-C, non–high-density lipoprotein cholesterol, and apolipoprotein B. Also, a greater proportion of the LCHF group had hypercholesterolemia relative to the SD group as per LDL-C and apolipoprotein B criteria. In the cohort subset, the LCHF group also showed higher levels of ketone bodies.

Notably, in the subset of the cohort followed up for an average of 11.8 years, individuals reporting an LCHF diet were 2.18 times more likely to have a MACE than participants reporting an SD (HR: 2.18, 95% CI: 1.39-3.43, *P* < 0.001) after adjusting for race/ethnicity, income, education, and CV risk factors (diabetes, body mass index, hypertension and smoking). Individuals reporting an LCHF diet with LDL-C levels ≥5 mmol/L (equivalent to ≥193.4 mg/dL) had the greatest ASCVD risk (HR: 6.68, 95% CI: 2.62-17.09).

Findings were confirmed in subgroup and sensitivity analyses when including participants on lipid-lowering therapy, using different definitions for carbohydrate restriction (<50 g/d or <100 g/d), and excluding individuals with diabetes. Results were confirmed for lipid profiles in participants with ≥2 dietary recalls, but the risk of ASCVD was attenuated with a smaller sample size.

Regarding genetic background, individuals with a high PRS reporting an LCHF diet demonstrated the highest LDL-C levels and the greatest proportion of individuals with hypercholesterolemia (32.3%). A significant interaction was reported between dietary pattern and PRS that confirmed individuals reporting an LCHF diet with a high PRS had higher LDL-C concentrations compared to participants with a nonhigh PRS.

These findings demonstrate associations between a self-reported LCHF dietary pattern, increased cholesterol and ketone body concentrations, and increased risk of MACE. These results remained significant after including individuals on lipid-lowering therapy, excluding individuals with diabetes, applying alternative definitions of carbohydrate restriction, and including only individuals with ≥2 dietary recalls. Also, individuals with a high PRS reporting an LCHF diet were more likely to exhibit hypercholesterolemia by LDL-C and apolipoprotein B standards. Of note, the LCHF diet definition used in this study does not represent a therapeutic ketogenic diet, as evidenced by ketone body concentrations above nutritional ketosis thresholds. This broadens generalizability to LCHF diets more likely to be consumed by the general population.

As the authors note, mixed results have been reported for the impact of LCHF diets on lipid levels. The heterogeneity of results may be due to variation in the quality and source of macronutrients in LCHF diets. Plant-based food sources tend to be higher in fiber, unsaturated fatty acids, micronutrients, and plant bioactives.^4^ They also differ in amino acid profiles relative to animal products and may interact with the gut microbiome to reduce CVD risk.^4^ However, LCHF diets often include animal products that are low in fiber and high in saturated fatty acids (SFA) that may increase LDL-C levels and raise ASCVD risk. Here, participants in the LCHF group reported consuming twice the energy from animal fat (33.2% vs 16.8%, respectively), greater energy from animal proteins (17.8% vs 10.2%, *P* < 0.001), and more energy from SFA (17.4% vs 11.2%) relative to the SD group. Current nutrition recommendations suggest limiting SFA and replacing SFA with unsaturated fatty acids to reduce CVD risk.^5^, ^6^, ^7^

These findings also underscore the variability in individual response to diet. For example, while lipid concentrations were mildly elevated in the LCHF group compared to the SD group, nearly double the proportion of participants in the LCHF group exhibited hypercholesterolemia. Yet, there was wide variation in lipid levels in the SD group. Additionally, individuals with a high PRS who reported an LCHF diet had the highest LDL-C concentrations and greatest proportion of individuals (32.3%) with hypercholesterolemia. Many people are unaware of their PRS and would not know if they are more likely to respond negatively to an LCHF diet, which may be an important consideration.

Individuals select dietary patterns for a variety of reasons. LCHF diets are common for weight and/or fat loss. However, in a meta-analysis of controlled feedings studies, LCHF diets were less effective in fat loss relative to low-fat diets, although the difference (16 g/d) was not clinically significant.^8^ Long-term adherence to an LCHF diet may be challenging. Being aware of the food choices available within a dietary pattern is important. While LCHF diets are low in added sugar and refined grains, they can limit nutrient-rich food groups such as fruits, legumes, whole grains, and some vegetables. Ultimately, this can lead to lower consumption of fiber, vitamins and minerals, and antioxidants that are linked to decreased disease risk.^7^ Quality of macronutrient consumption should underscore dietary pattern selection, and counseling with a registered dietitian can be helpful when selecting a dietary pattern, particularly for clinical populations.

This study contributes compelling results in a large, prospective cohort study. Several limitations should be noted. This was an observational research study that does not infer causation. Studying dietary intake is inherently complex as nutrients are not eaten in isolation but within a matrix of foods, which affects digestion and absorption, and there are interindividual differences in these factors. Assessing dietary intake is also difficult. Participants self-reported dietary intake with at least 1 24-hour recall and findings were similar when including only participants with ≥2 diet recalls. Three 24-hour dietary recalls are recommended to capture habitual dietary intake. The authors controlled for obesity (assessed by body mass index) and diabetes status, and conducted analyses excluding individuals with diabetes, but residual confounding may remain by including participants with increased CVD risk. Finally, the majority of this cohort was females of White British descent, and generalizability may be limited to other racial/ethnic groups.

Congratulations to the authors on their work and important contributions to the literature on dietary patterns and CVD. While the diets and individual response to LCHF diets can vary greatly, these findings caution against self-prescription to an LCHF diet, which was associated with increased levels of atherogenic lipids and higher risk of MACE. Future research may investigate these associations in more diverse populations, test if associations differ by sex, and determine factors contributing to the heterogeneity in response to dietary patterns.

## Funding support and author disclosures

Funding provided by 10.13039/100000968American Heart Association grant 23SFRNPCS1063854 and 10.13039/100000002National Institutes of Health grant T32CA093423 and Susan G. Komen Foundation grant CTA241184399. The authors have reported that they have no relationships relevant to the contents of this paper to disclose.
